# Epidemiological, Entomological, and Climatological Investigation of the 2019 Dengue Fever Outbreak in Gewane District, Afar Region, North-East Ethiopia

**DOI:** 10.3390/insects13111066

**Published:** 2022-11-18

**Authors:** Wondemeneh Mekuriaw, Solomon Kinde, Bezabih Kindu, Yibeyin Mulualem, Girma Hailu, Araya Gebresilassie, Chalachw Sisay, Fitsum Bekele, Hiwot Amare, Mesfin Wossen, Adugna Woyessa, Chad L. Cross, Louisa A. Messenger

**Affiliations:** 1 Ethiopian Public Health Institute, Addis Ababa 1242, Ethiopia; 2 College of Computational and Natural Science, Addis Ababa University, Addis Ababa 1000, Ethiopia; 3 National Meteorological Agency, Addis Ababa P.O. BOX 1090, Ethiopia; 4 Department of Epidemiology and Biostatistics, School of Public Health, University of Nevada, Las Vegas, NV 89154, USA; 5 Department of Disease Control, Faculty of Infectious and Tropical Diseases, London School of Hygiene and Tropical Medicine, London WC1E 7HT, UK; 6 Department of Environmental and Occupational Health, School of Public Health, University of Nevada, Las Vegas, NV 89154, USA

**Keywords:** dengue fever, arbovirus, *Aedes aegypti*, Ethiopia, climate change, outbreak

## Abstract

**Simple Summary:**

Dengue Fever (DF) is a viral disease transmitted by mosquitoes throughout tropical, sub-tropical, and some temperate climates worldwide. DF ranges in severity from mild flu-like symptoms to severe joint and muscle pain and even death from severe dengue. In the past 50 years, DF incidence has increased 30-fold, with 3.9 billion people in 129 countries currently considered at-risk annually. DF has been circulating on the African sub-continent for decades, but actual cases are highly under-reported due to their non-specific symptomology and overlapping distributions with other febrile illnesses, notably malaria and pneumonia. Ethiopia has been experiencing annual DF outbreaks since 2013. This study describes key epidemiological, entomological, and climatological features of the first DF outbreak in Gewane District, Afar Region, Ethiopia, in 2019. A total of 1185 DF cases were identified, mostly among individuals aged 15–49, experiencing fever, headache, and joint pain; there was no recorded death. Mosquito species that transmit DF (*Aedes aegypti*) were found breeding in plastic tanks, tires, and plastic or metal buckets/bowls in and around DF patients’ homes. High rainfall in July 2019 and long-term storage of uncovered, stagnant drinking water by community members were implicated in this outbreak. The study results emphasize the need for control activities targeting *Ae. aegypti* and improved entomological surveillance to prevent future DF outbreaks in this part of Ethiopia.

**Abstract:**

Dengue Fever (DF) is an important arthropod-borne viral infection that has repeatedly occurred as outbreaks in eastern and northeastern Ethiopia since 2013. A cross-sectional epidemiological outbreak investigation was carried out from September to November 2019 on febrile patients (confirmed malaria negative) who presented with suspected and confirmed DF at both public and private health facilities in Gewane District, Afar Region, northeastern Ethiopia. Entomological investigation of containers found in randomly selected houses belonging to DF-positive patients was undertaken to survey for the presence of *Aedes* larvae/pupae. A total of 1185 DF cases were recorded from six health facilities during the 3-month study period. The mean age of DF cases was 27.2 years, and 42.7% of cases were female. The most affected age group was 15–49 years old (78.98%). The total case proportions differed significantly across age groups when compared to the population distribution; there were approximately 15% and 5% higher case proportions among those aged 15–49 years and 49+ years, respectively. A total of 162 artificial containers were inspected from 62 houses, with 49.4% found positive for *Aedes aegypti* larva/pupae. *Aedes* mosquitoes were most commonly observed breeding in plastic tanks, tires, and plastic or metal buckets/bowls. World Health Organization entomological indices classified the study site as high risk for dengue virus outbreaks (House Index = 45.2%, Container Index = 49.4%, and Breteau Index = 129). Time series climate data, specifically rainfall, were found to be significantly predictive of AR (*p* = 0.035). Study findings highlight the importance of vector control to prevent future DF outbreaks in the region. The scarcity of drinking water and microclimatic conditions may have also contributed to the occurrence of this outbreak.

## 1. Introduction

Dengue Fever (DF) is an important mosquito-borne viral infection that has repeatedly occurred as outbreaks in eastern and northeastern Ethiopia since 2013 [1,2]. This infection causes flu-like symptoms and occasionally develops into a potentially lethal complication called severe dengue or dengue hemorrhagic fever (DHF), especially following repeated infections with different viral serotypes. In the last two decades, dengue cases reported to the World Health Organization (WHO) have risen by more than eightfold, from 505,430 cases in 2000 to over 2.4 million in 2010 and 4.2 million in 2019 [3]. Furthermore, the geographical range of dengue virus has expanded to new countries, as well as from urban to rural settings, largely due to climate change [4,5,6].

The actual number of dengue cases is underreported, and many cases are misclassified. A recent estimate indicated that 390 million (95% credible interval 284–528 million) dengue infections occur per year, of which 96 million (67–136 million) manifest clinically (with any severity of disease) [3]. Approximately 22,000 die from severe dengue annually [7]. The WHO has estimated that 3.9 billion people in 129 countries are at risk of dengue infection each year [3]. With early diagnosis and proper management, the case-fatality rate (CFR) of DHF is generally under 1%, but the CFR may rise to over 10% once shock develops [8]. 

Dengue viruses are transmitted by the bite of female *Aedes* mosquitoes. *Aedes aegypti* is the principal vector of dengue viruses in almost all countries worldwide [9,10]; this vector species prefers to rest indoors and feed on human blood during daylight hours [11], particularly during the early morning and evening [12]. The eggs of this mosquito can withstand dry weather conditions for multiple months and hatch when exposed to water [11,13,14,15]. Once emerged, adult *Aedes* mosquitoes can bite multiple times before oviposition [16]. *Ae. africanus*, *Ae. albopictus* [17], and *Ae. luteocephalus* also act as potential arbovirus vectors in Africa [10].

The extrinsic incubation period of the virus in the mosquito gut is highly dependent on ambient air temperature; viral development takes ~8–12 days when the ambient air temperature is between 25 and 28 °C [18,19]. Once bitten by an infectious mosquito, the intrinsic incubation period of DF in people is ~4–10 days [20]. High temperature, humidity, and extended rainfall are significantly associated with increased incidence of DF [21]. Other notable risk factors for dengue infection include those that facilitate mosquito breeding, such as having uncovered, stagnant water in containers (e.g., buckets, drums, tires, pots, etc.), and those that increase vector–human contact, for example, lack of a door or window screens and no use of personal protective measures (e.g., repellents) [22,23,24].

In Ethiopia, DF was diagnosed among returning travelers but was never reported as a local outbreak until September 2013 [25]. The first major outbreak occurred in Dire Dawa city (East Ethiopia), with a total of 11,409 cases [1]. The following year, in 2014, another outbreak was reported in Dire Dawa, as well as in Godey Town, Somali Region (South-East Ethiopia) and in Ada’ar Woreda, Afar Region (North Ethiopia) [2,24]. Subsequently, annual outbreaks have been observed in Godey Town and Dire Dawa [26,27]. Circulating dengue infection has also been identified in Humera, Tigray Region, Metema, Amhara Region, Arba Minch, Southern Nations, Nationalities, and People Region, Gondar, Amhara Region, and Borena Zone, Oromia Region, by the presence of anti-DENV IgG/IgM antibodies in febrile patients [28,29,30,31], indicating that the true distribution of DF in Ethiopia is highly underestimated. More recently, in 2017, DF emerged in Kabridahar Town, Korahey Zone (Eastern Ethiopia), affecting more than 100 individuals [24], and in 2019, for the first time in Gewane District, Afar Region (North-East Ethiopia).

To date, there is still a severe paucity of data describing the epidemiology of DF and its association with climatic factors in Ethiopia. Moreover, DF incidence is likely severely underreported in Ethiopia due to the lack of Integrated Disease Surveillance and Response (IDSR) reporting requirements for DF, limited regional and national laboratory capability to confirm cases and the remoteness of many endemic areas. The aim of the present study was to investigate the epidemiological, entomological, and climatological parameters associated with the 2019 DF epidemic in northeastern Ethiopia. 

## 2. Materials and Methods

### 2.1. Study Area

**Figure 1:**
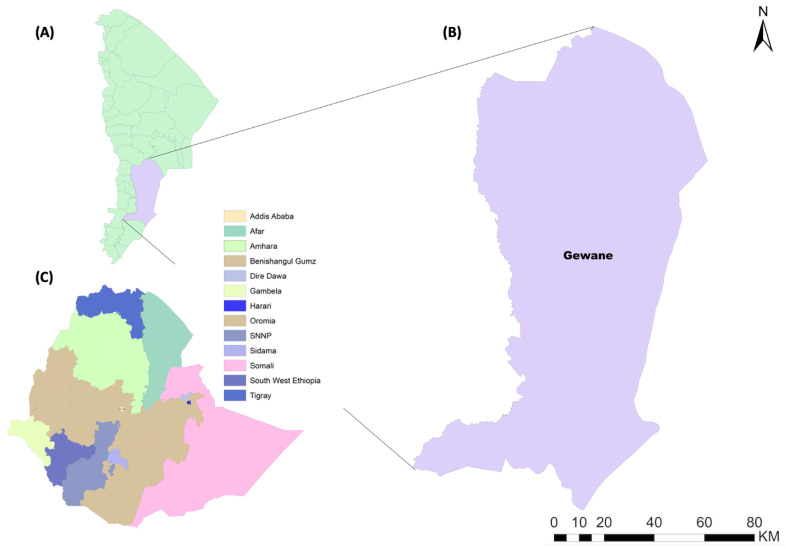
Map of Afar Region (**A**) and the study area, Gewane District (**B**), relative to all Regional States in Ethiopia (**C**).

**Figure 2:**
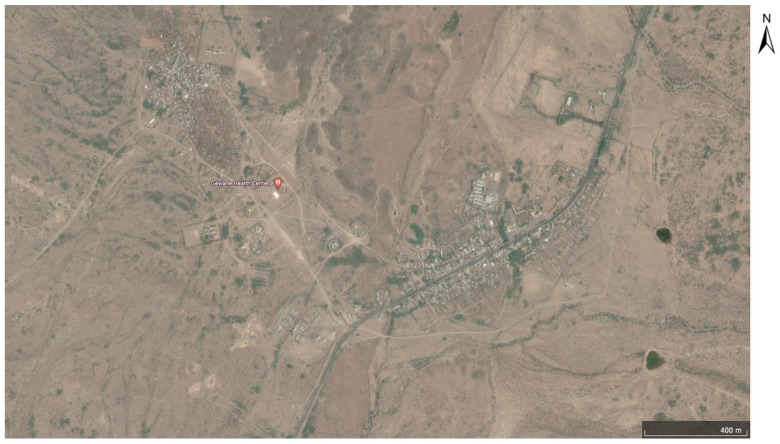
Satellite image of Gewane District, indicating the main health center. Source: Google Earth Pro. [Gewane District]. Retrieved 31 October 2022.

This study was conducted in Gewane District (10.1496 N, 40.66894 E), an urban/peri-urban area that is administratively located in Zone 3, Afar Region, Ethiopia. Gewane District is found along the highway from Addis Ababa to Djibouti, 372 km North-East of Addis Ababa and 226 km from Semera, the capital of Afar Regional State (Figure 1 and Figure 2). The district is located at a low-lying elevation, 618 m above sea level. Gewane District has a total of 22,322 inhabitants, of which 11.3% (*n* = 2522) are children under five years of age. The district has a pipeline water source; however, water was available only once per week.

### 2.2. Study Design, Period and Population

The present study employed a cross-sectional design and was performed between September and November 2019. All of the study participants were confirmed free of malaria infection using either microscopy or a rapid diagnostic test (RDT). Confirmed (by quantitative real-time PCR; qRT-PCR) and epidemiologically linked DF cases at both public and private healthcare facilities in Gewane District were considered for this study. A line list of every case identified during the study period was collected. Demographic information, including age and sex, as well as other data, including place of residence and date of onset of signs and symptoms, were carefully retrieved from records. In addition, data on the period of admission, admission status, laboratory results, and final patient outcome were collated.

### 2.3. Entomological Investigation

An entomological investigation of the containers for the presence of *Aedes* larvae and pupae was conducted in the houses and premises belonging to DF patients and family members that were selected randomly from the complete DF patient list. The mosquito samples were collected from infested containers with a plastic cup, pipette, and dipper, and larvae/pupae were reared to the adult stage for identification using morphological identification keys [32]. The entomological indices were interpreted according to WHO guidelines; high risk of dengue virus transmission was when the House Index (percentage of houses found positive for mosquito larvae or pupae; HI) >35%, Breteau Index (number of containers found positive for mosquito larvae or pupae; BI) >50, or Container Index (percentage of containers found positive for mosquito larvae or pupae; CI) >20%. Low risk was when HI < 4%, BI < 5, or CI < 3%.

### 2.4. Laboratory Investigation

To confirm the aetiological agent of the outbreak, blood specimens (3–5 mL) were collected from 12 suspected DF cases during the cross-sectional survey and transported to the National Influenza and Arbovirus Laboratory at the Ethiopian Public Health Institute (EPHI), Addis Ababa. The extraction of viral RNA was performed from serum using the QIAamp Viral RNA Mini Kit (Qiagen, Germany) and tested for dengue virus by qRT-PCR. A dengue-virus-specific primer and the Agpath-ID™ one-step qRT-PCR kit (ThermoFisher Scientific, London, UK) were used to detect the presence of dengue virus. Appropriate negative and positive controls were tested in parallel. The reaction conditions for the qRT-PCR were reverse transcription at 45 °C for 10 min, denaturation at 95 °C for 10 min, followed by 45 cycles of denaturation at 95 °C for 15 s and annealing/extension at 55 °C for 1 min. Fluorescence was measured at the annealing/extension step and recorded as the cycle threshold (CT) value. The specimens were considered positive if the CT value was < 40, indeterminate if the CT was ±40, and negative if the CT value was undetermined. Similar laboratory procedures were used for testing the blood specimens for other arboviruses (Chikungunya, Yellow Fever, Rift Valley Fever, and West Nile Virus) using viral-specific primers and appropriate negative and positive controls.

### 2.5. Climatological Data

Quality-controlled climate data for Gewane District were obtained from the National Meteorological Agency (NMA) of Ethiopia. Three-year rainfall, temperature, and relative humidity were analyzed for the study area.

### 2.6. Data Analysis

The entomological and epidemiological data were entered, cleaned, and analyzed using SAS and R. The attack rate (AR; proportion of an at-risk population that contracts the disease during a specified period) was calculated for age-group comparisons and for time series analyses based on the date of disease onset reported in Epi-Link. The risk of DF in the study district was interpreted according to entomological indices set by the WHO [33]. Population projections for Afar Region, determined by the central statistics agency, were used to compute the population at-risk in each age group [34].

The exact binomial test was used to compare the proportion of total positive cases between sexes and between container positivity rates, with a null expectation of a 50:50 distribution. Chi-square analyses with Cramer’s V effect sizes were utilized to compare sexes among age groups, as well as to compare sex distributions of cases to the underlying population distribution. For the entomological investigation, the Fisher–Freeman–Halton test was used to examine potential associations between containers and the frequency counts of positive tests. Owing to the relatively small sample size for the container study, we utilized the 99% upper bound of *n* = 10,000 Monte Carlo resamples to obtain an estimated *p*-value.

We examined the AR and climatological data using a time series approach. To understand potential historical climate trends, we first calculated distance-based energy correlations (r_d_) among years 2017–2019 for average temperate, relative humidity, and rainfall using *n* = 10,000 Monte Carlo replicates to estimate the *p*-values. This group of statistics is specifically designed to measure differences in the distance of distributions of random vectors and accommodates testing distributional similarities and differences without making parametric assumptions that are associated with other methods, hence making them more appropriate for analyzing time-trended data [35,36,37,38]. We also utilized autoregression to investigate the potential of climate variables to estimate 2019 AR values through time.

## 3. Results

### 3.1. Epidemiological Investigation

**Figure 3:**
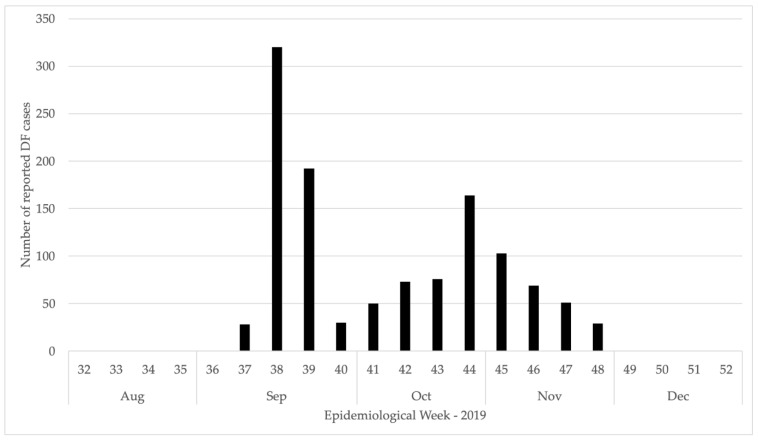
Distribution of reported DF cases by their date of onset, from September to November 2019 in Gewane District, Afar Region, Ethiopia (*n* = 1185).

A total of 1185 DF cases were recorded between September and November 2019; no other DF cases were reported in 2019 (between January and September or in December), nor in 2017 or 2018. Of these cases, twelve had serum samples taken, and six tested positive for DF by qRT-PCR at EPHI, with the remaining 1179 defined through Epi-Link. DF patients originated from ten kebeles: Bida (*n* = 2; 0.17%), Bireforo (*n* = 41; 3.5%), Gebayabora (*n* = 60; 5.1%), Geliladora (*n* = 239; 20.2%), Gewane (*n* = 736; 62.1%), Kedabada (*n* = 6; 0.51%), Keroma (*n* = 2; 0.17%), Meteka (*n* = 72; 6.08%), Ourafita (*n* = 4; 0.34%), and Yigilla (*n* = 23; 1.9%); and presented at six health facilities: Bireforo health center (*n* = 42), Geliladora hospital (*n* = 246), Gewane health center (*n* = 416), Meteka hospital (*n* = 4), Yigilla hospital (*n* = 15), and private clinics (*n* = 462). The index case was reported from Bireforo health center, with the onset of symptoms on 13 September 2019. The initial peak of the outbreak occurred from 16 to 29 September 2019, with a secondary peak between 28 October and 10 November 2019 (Figure 3). The six positive serum specimens were collected from Gewane Health Clinic during WHO epidemiological week 38 from five males (aged 22–40) and one female (aged 40). The six negative specimens were also collected from Gewane Health Clinic during WHO epidemiological week 38 from five males (aged 30–38) and one female (aged 24). None of the twelve serum samples were positive for any other arboviruses tested (Chikungunya, Yellow Fever, Rift Valley Fever, and West Nile Virus).

The mean and median ages of DF cases were 27.2 and 26 years old, respectively. A total of 42.7% of the cases were female, which was statistically less than males (exact binomial *p* < 0.001; 95% CI: 39.8%, 45.5%). However, the distribution of cases between sexes across age groups was equitable (χ^2^ = 1.56, ϕ = 0.036, *p* = 0.669). The total case proportions differed significantly across age groups when compared to the population distribution (χ^2^ = 260.20, V = 0.271, *p* = < 0.001), and this was true when examining both the distribution of female cases (χ^2^ = 116.65, ϕ = 0.278, *p* < 0.001) and male cases (χ^2^ = 148.80, ϕ = 0.270, *p* < 0.001). Interestingly, there were approximately 15% and 5% higher case proportions among those aged 15–49 years and 49+ years, respectively, compared to their representation in the population, with subsequently lower representation among the other age groups. The AR was highest in the 49+ age group (134.2), followed by 15–49 years old (65.7) per thousand (Table 1). All suspected cases (*n* = 1185) reported experiencing fever, headache, and joint pain. No patients had a travel history to a dengue-endemic area. There were no recorded deaths associated with DF in Gewane District during the study period. 

### 3.2. Entomological Investigation

**Figure 4:**
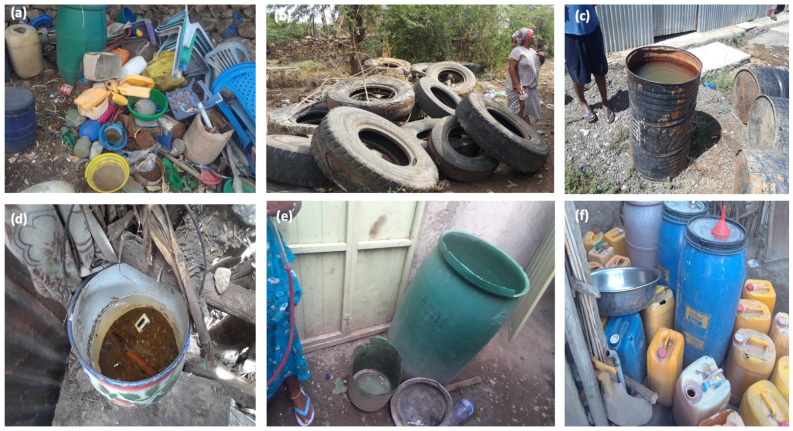
Typical *Aedes* breeding sites identified in Gewane District, Ethiopia, 2019: (**a**) plastic buckets/bowls; (**b**) rubber tires; (**c**) metal drum; (**d**) pots; (**e**) large plastic tanks; and (**f**) small plastic tanks.

A total of 162 artificial containers were inspected at 66 and 96 houses from Gubi Gewane and Gewane Town-01 kebele, respectively (Figure 4). Of these, 80 containers (49.4%) from 28 houses were positive for *Aedes* larva/pupae, representing a statistical 50:50 ratio (exact binomial *p* = 0.937, 95%CI: 41.4%, 57.3%). Two thousand, four hundred and nine immature stages were reared to adulthood; of these, 1978 emerged and were identified as *Ae. aegypti* (*n* = 792), *Anopheles* spp. (*n* = 534) and *Culex* spp. (*n* = 652) by morphology. *Ae. aegypti* were most commonly observed breeding in plastic tanks, tires, and plastic or metal buckets/bowls (Table 2). There was no statistical association between container type and positivity status (χ^2^ = 6.47, ϕ = 0.198, *p* = 0.082), though container samples were not distributed equitably.

To estimate the potential dengue outbreak risk, the WHO entomological indices (house index; HI, container index; CI and Breteau index; BI) were calculated. During this investigation, the entomological indices were very high, with a HI of 45.2% (WHO high-risk threshold = HI > 35%), a CI of 49.4% (WHO high-risk threshold = CI > 20%), and a BI of 129 (WHO high-risk threshold = BI > 50).

### 3.3. Climatological Data

To examine potential climate irregularities in 2019 that may have contributed to the outbreak, we examined historical data between 2017 and 2018. The mean monthly rainfall was significantly similar among all years (2017 vs. 2018, r_d_ = 0.175, *p* = 0.0176; 2017 vs. 2018, r_d_ = 0.233, *p* = 0.002; 2018 vs. 2019, r_d_ = 0.250, *p* = 0.001); however, the maximum monthly rainfall in 2019 occurred earlier in the year (i.e., July) compared to 2017 and 2018 (i.e., August). Relative humidity was significantly correlated for 2017 vs. 2019 (r_d_ = 0.305, *p* < 0.001) but not for the other years (2017 vs. 2018, r_d_ = 0.113, *p* = 0.104; 2018 vs. 2019, r_d_ = 0.117, *p* = 0.104). Additionally, the temperature distributions were highly correlated among all years (2017 vs. 2018, r_d_ = 0.701, *p* < 0.001; 2017 vs. 2018, r_d_ = 0.790, *p* < 0.001; 2018 vs. 2019, r_d_ = 0.616, *p* < 0.001); although the shape trend of the distributions was the same, mean temperatures were on average 7 °C lower in 2017. 

**Figure 5:**
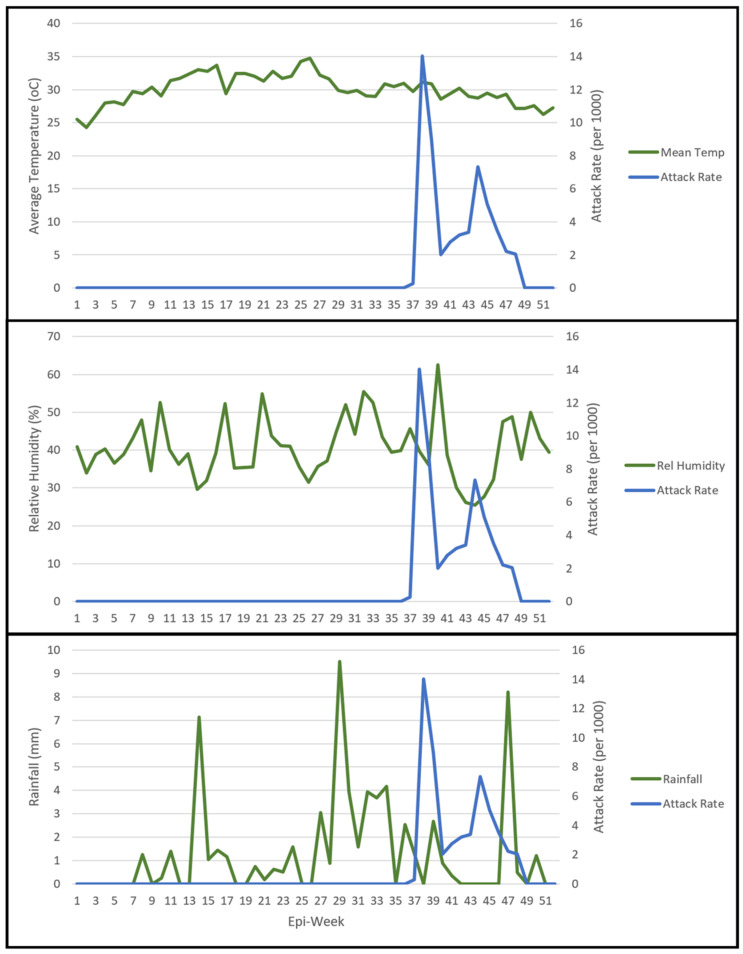
Climate data (mean temperature, relative humidity, and rainfall) plotted with weekly attack rate by WHO Epi-week for Gewane District for 2019.

The examination of the 2019 climate data compared specifically to weekly ARs suggested some potentially interesting findings (Figure 5). The mean temperature was relatively stable over time in Gewane District, though it decreased during the period of the outbreak. Both relative humidity and rainfall were quite variable. In 2019, the highest weekly cumulative rainfall was recorded in WHO Epi-week 29. The present DF outbreak emerged in Epi-week 37, indicating a lag phase of approximately 8 weeks. Additionally, the outbreak occurred during drier periods, with the highest ARs specifically during Epi-weeks coinciding with demonstrable drops in relative humidity (Figure 5).

A time series model was estimated based on 2019 climate data. Using the time lags suggested above to offset the raw data, weekly AR could be estimated using rainfall (β = 0.724, *p* < 0.001), relative humidity (β = 0.088, *p* < 0.029), and average temperature (β = −0.302, *p* < 0.040). However, the large period of no infections prior to Epi-week 37 and only utilizing data from the period of infections through the end of 2019 resulted in only rainfall being a significant predictor of AR (β = 0.9753, *p* = 0.0352). 

### 3.4. Outbreak Response Activities 

Due to the scarcity of drinking water in Gewane District, community members regularly collect water in many plastic containers to use over several weeks. Most of the containers were old and did not have covers, thus providing abundant sites for mosquito oviposition. There were a number of old, discarded tires, which formed another source of stagnant water. As part of the outbreak response, social mobilization and awareness of disease control strategies, particularly environmental manipulation (removing, altering, or recycling non-essential containers, which were serving as vector habitats), was undertaken using megaphones, leaflets, and during house-to-house active case searches.

## 4. Discussion

To reduce the risk of future DF and other arboviral disease outbreaks in Ethiopia through appropriate policy recommendations, it is crucial to understand the key epidemiological, entomological, and climatological features of previous outbreaks. This study reports the first DF outbreak in Gewane District, Afar Region, Ethiopia, affecting more than one thousand individuals over a 3-month period in 2019. The epidemiological characteristics of this outbreak were consistent with previous studies, which also documented more DF cases among the male population (57.3%) due to gender, cultural, and behavioral differences [24,26,28,39]. *Aedes* mosquitos may bite outside during the daytime when many female community members are commonly inside the house and/or may have their entire body covered, according to Islamic Law, providing a defensive layer from mosquito bites. Other reports from Ethiopia have confirmed that daily wearing of short sleeve T-shirts was strongly associated with a higher likelihood of DF infection compared to those in full dress [24]. The median age of DF cases in Gewane District was 26 years old, with a 15% higher proportion of cases in those aged 15–49 years, which also aligns with other DF outbreaks in Ethiopia [26], where younger age groups spent more time outdoors due to economic or leisure pursuits and were, therefore, at higher risk of vector contact. While not directly assessed during this study, poor knowledge of DF prevention in other parts of Ethiopia, among both community members and healthcare providers, has been identified as a significant barrier to the prevention of future outbreaks, particularly awareness of the importance of emptying or covering containers that can be used as *Aedes* breeding sites [2,27,29]. It can be assumed that in areas similar to Gewane District, where DF has emerged for the first time, community education may be especially low, and initiatives to increase nationwide knowledge of DF and other arbovirus control are needed. Since this present study, further DF outbreaks have occurred in the Somali Region of Ethiopia (Warder Woreda of Dolo Zone and Dolo Ado Woreda of Liban Zone) during 2021 [40], indicating that the geographical range of DF in Ethiopia is expanding on an almost yearly basis.

The entomological findings of the study classified Gewane District as high risk for DF transmission, with HI = 45.2%, CI = 49.4%, and BI = 129; entomological indices were similar to those reported from other recent DF outbreaks in Kabridahar District, Somali Region in 2017 [24]. However, these criteria suffer from several limitations, particularly from differences in container productivity, and should be interpreted with caution; none of the indices account for relative adult *Ae. aegypti* yield per infested breeding site nor confer a measure of transmission risk per person [41]. Given the predominance of *Ae. aegypti* breeding in this urban/peri-urban area, our results strongly emphasize the need for regional vector control activities, improved entomological surveillance and strengthening of laboratory capacity to prevent future DF outbreaks. As part of this outbreak response, the study team undertook the removal of potential *Aedes* breeding sites within the community and increased awareness of DF control strategies. 

High rainfall in July 2019 was a significant predictor of AR in this study, presumably increasing the number of available breeding sites (i.e., water-filled containers) and overall adult vector population density. However, the relationship between rainfall and DF incidence is not absolute. Some studies have demonstrated that heavy rainfall can lower dengue virus transmission by reducing the daily survival rate of *Ae. aegypti* [42,43,44] or by causing breeding sites to overflow and wash away developing larvae (“flushing”) [45,46]. Such phenomena may explain why no DF was observed in Gewane District following the occurrence of high rainfall in Epi-week 14 of 2019, although it is unclear exactly which microclimatic conditions were sufficient to enable viral transmission in Epi-week 37 compared to earlier in this year or in the two preceding years. In this study, the lag phase was approximately 8 weeks, which was consistent with other DF surveillance data, demonstrating that precipitation had the highest transmission risk with 8–15 lag weeks [47]. In addition to climatic factors, the lack of piped water and, therefore, long-term storage of uncovered, stagnant drinking water by community members also likely contributed to this outbreak [48]. As water shortage is a prevailing issue in Gewane District, necessitating permanent use of water tanks, additional environmental manipulation measures, such as container covers, could be considered [49]. To improve prospective DF vector control, during other outbreaks in Ethiopia, the distribution of long-lasting insecticidal nets (LLINs) has been shown to be protective because some residents slept under nets early in the morning and during the daytime in response to harsh weather conditions [2,24,28]. Furthermore, the use of LLIN material as window curtains has yielded contrasting evidence to reduce DF transmission in parts of Latin America, but the efficacy of these tools has yet to be established for DF control in sub-Saharan Africa [50,51,52]. 

This study had several limitations. Community-level serological diagnosis was not possible to confirm all suspected cases or to screen for asymptomatic carriers. Because the epidemiological investigation was based on clinical data and not all laboratory specimens were confirmed as DF-positive, it is possible that some patients were infected with other acute non-malarial febrile illnesses. This study lacked the resources to identify which DENV serotype(s) was responsible for this outbreak; however, the low CFR (no deaths reported during the outbreak) may either indicate a recent introduction of DF into this area, with few prior infections in the population, or re-emergence of the same circulating DENV serotypes, which would not elicit severe dengue symptoms. Given the geographical proximity of Gewane District to other DF outbreaks (Dire Dawa: 376 km), it is quite probable that human movement also played a role in the occurrence of this outbreak. The extraordinarily high AR in the 49+ age group should be interpreted cautiously, as this is likely a function of the small denominator, which only represents 2.9% of the total population, and is not an indication of increased risk among this group *per se*. Time series analyses of outbreaks are subject to many caveats, not least among them is limited data. For this reason, we interpret the time series analyses as preliminary and hypothesis-generating. 

## 5. Conclusions

DF is an important arthropod-borne viral infection that has repeatedly occurred as outbreaks in eastern and northeastern Ethiopia since 2013. A cross-sectional epidemiological outbreak investigation was carried out from September to November 2019 on febrile patients (confirmed malaria negative) who presented with suspected and confirmed DF at six health facilities in Gewane District, Afar Region, northeastern Ethiopia, identifying a total of 1185 DF cases. The mean age of DF cases was 27.2 years, and 42.7% of the cases were female. The most affected age group was 15–49 years (78.98%). However, the attack rate (AR) was highest in the 49+ age group (134.2). A total of 162 artificial containers were inspected from 62 houses, with 49.4% found positive for *Ae. aegypti* larva/pupae, mainly breeding in plastic tanks, tires, and plastic or metal buckets/bowls. High rainfall in July 2019, lack of piped water and, therefore, long-term storage of uncovered, stagnant drinking water by community members were implicated in this outbreak. Since this present study, further DF outbreaks have occurred in the Somali Region of Ethiopia (Warder Woreda of Dolo Zone and Dolo Ado Woreda of Liban Zone) during 2021. The study results strongly emphasize the need for nationwide control activities, targeting arbovirus vector species, and improved entomological surveillance to prevent future DF outbreaks in this part of Ethiopia.

## Figures and Tables

**Table 1:** Age-specific DF attack rates in Gewane District, Afar Region, Ethiopia (*n* = 1185).

Age Category	Female (%) ^a^	Male (%) ^a^	Total Cases (%) ^a^	Population (2019) ^a^	Attack Rate (per 1000)
**<5**	12 (2.4)	21 (3.1)	33 (2.8)	2522 (11.2)	13.1
**5–14**	60 (11.9)	70 (10.3)	130 (11.0)	4920 (22.0)	26.4
**15–49**	394 (78.0)	542 (79.7)	936 (79.0)	14,239 (63.8)	65.7
**49+**	39 (7.7)	47 (6.9)	86 (6.9)	641 (2.9)	134.2
**Total**	505	680	1185	22,322	53.1

^a^ The percentage shown is out of total cases per column.

**Table 2:** Proportions of containers found positive with either *Aedes* larvae or pupae in Gewane District, Ethiopia, 2019.

Container	Negative (%) ^a^	Positive (%) ^a^	Total Samples (%) ^a^	Positivity Rate (%)
**Plastic or Metal Buckets/Bowls**	2 (2.4)	10 (12.5)	12 (7.4)	83.3
**Clay Jars**	2 (2.4)	2 (2.5)	4 (2.5)	50.0
**Plastic Tanks**	42 (51.2)	40 (50.0)	82 (50.6)	48.8
**Tires**	36 (43.9)	28 (35.0)	64 (39.5)	43.8
**Total**	82	80	162	49.4

^a^ The percentage shown is out of total cases per column.

## Data Availability

The data sets generated and/or analyzed during the current study are available from the corresponding authors on reasonable request.
